# MCE: Medical Cognition Embedded in 3D MRI feature extraction for advancing glioma staging

**DOI:** 10.1371/journal.pone.0304419

**Published:** 2024-05-31

**Authors:** Han Xue, Huimin Lu, Yilong Wang, Niya Li, Guizeng Wang

**Affiliations:** 1 School of Computer Science and Engineering, Changchun University of Technology, Changchun, Jilin, China; 2 Key Laboratory of Symbolic Computation and Knowledge Engineering of Ministry of Education, Jilin University, Changchun, Jilin, China; 3 Jilin Provincial Smart Health Joint Innovation Laboratory for the New Generation of AI, Changchun, Jilin, China; 4 The First Hospital of Jilin University, Changchun, Jilin, China; 5 School of Mathematics and Statistics, Changchun University of Technology, Changchun, Jilin, China; Union Hospital, Tongji Medical College, Huazhong University of Science and Technology, CHINA

## Abstract

In recent years, various data-driven algorithms have been applied to the classification and staging of brain glioma MRI detection. However, the restricted availability of brain glioma MRI data in purely data-driven deep learning algorithms has presented challenges in extracting high-quality features and capturing their complex patterns. Moreover, the analysis methods designed for 2D data necessitate the selection of ideal tumor image slices, which does not align with practical clinical scenarios. Our research proposes an novel brain glioma staging model, Medical Cognition Embedded (MCE) model for 3D data. This model embeds knowledge characteristics into data-driven approaches to enhance the quality of feature extraction. Approach includes the following key components: (1) Deep feature extraction, drawing upon the imaging technical characteristics of different MRI sequences, has led to the design of two methods at both the algorithmic and strategic levels to mimic the learning process of real image interpretation by medical professionals during film reading; (2) We conduct an extensive Radiomics feature extraction, capturing relevant features such as texture, morphology, and grayscale distribution; (3) By referencing key points in radiological diagnosis, Radiomics feature experimental results, and the imaging characteristics of various MRI sequences, we manually create diagnostic features (Diag-Features). The efficacy of proposed methodology is rigorously evaluated on the publicly available BraTS2018 and BraTS2020 datasets. Comparing it to most well-known purely data-driven models, our method achieved higher accuracy, recall, and precision, reaching 96.14%, 93.4%, 97.06%, and 97.57%, 92.80%, 95.96%, respectively.

## Introduction

According to the World Health Organization (WHO) [1], brain tumors pose a significant threat to global health and quality of life. Brain tumors are tumors that affect the central nervous system, characterized by abnormal proliferation of cells. They are categorized from Grade I to Grade IV based on their growth rate [2]. Specifically, Grade I and Grade II gliomas are classified as Low-Grade Gliomas (LGG), while Grade III and Grade IV gliomas classified as High-Grade Gliomas (HGG) [3]. Grade IV glioblastoma (GBM) is one of the most lethal and easily recurrent malignant solid tumors, accounting for 57% of all neuroglial tumors and 48% of primary central nervous system malignancies. The median survival period is usually no more than 2 years [4]. Brain Magnetic Resonance Imaging (MRI), as a non-invasive medical imaging technique, can clearly visualize the precise location, size, and morphology of tumors [5]. It is widely used for diagnosis and monitoring brain tumors. However, to achieve accurate assessment and staging of brain tumors through MRI images, radiologists must possess extensive experience and exceptional skills [6]. Furthermore, the training of radiologists in this specialized domain necessitates a substantial investment of time and financial resources. With the increasing volume of brain tumor image datasets, it becomes even more susceptible to subjective bias and human errors during the assessment [7, 8].

In recent years, deep learning algorithms such as Convolutional Neural Networks (CNN) have made revolutionary breakthroughs in the analysis of brain glioma MR images, providing new opportunities for the automated detection and classification of brain tumor MRI data [9]. These algorithms can learn complex features from MRI data and perform remarkably well on classification and staging tasks [10–12]. However, there are also some limitations: (1) Due to the scarcity of MRI data and the imbalance of categories, MRI analysis in data-driven deep learning algorithms lacks the guidance of prior knowledge and domain-specific rules. This can lead to excessive redundancy in automatically learned features and patterns, thereby affecting the model’s generalization ability [13]. (2) While deep features can effectively capture high-level abstract information in images, there are certain limitations in expressing specific features relevant to the staging of gliomas, such as texture, morphology, and grayscale distribution. The acquisition of texture and shape features requires meticulous texture analysis, contour extraction, and geometric measurements. (3) Researchers often observe that models mistakenly identify irrelevant features in images (e.g., artifacts, vignetting, background, etc.) as decision cues. In other words, models infer results based on non-relevant features and exhibit a certain degree of regularity [14]. This is a manifestation of the model’s excessive reliance on the specificity of training data.

To address the issues mentioned above, we propose a new method in this paper that incorporates external knowledge rules from the perspective of feature acquisition. We have designed the MCE model to enhance the quality of feature extraction. Additionally, given the limitation of 2D-based analysis methods necessitate the use of optimal tumor image slices, which may not align with clinical practice, our approach is thoughtfully tailored for 3D brain glioma MRI images. Specifically:

1. Designing different modules allows the MCE model to capture and learn different patterns and modes present in the data, including the attention patterns of doctors, radiomic information, and diagnostic knowledge. The combination of multiple modules effectively reduces the random fluctuations caused by the high complexity of a single model, thus mitigating overfitting issues and enhancing the model’s generalization capability.
2. Imitating the attention pattern of physicians during image interpretation—focusing on the characteristic regions of different sequence images. The paper designs, at both the model and strategy levels, to mimic the real process of physicians interpreting medical images, constructing two feature extraction models, Multi- Sequence Attention Neural Network (MSANN) and Clinical Learning Neural Network (CLNN).
3. Introduced the Radiomics module, which explores information such as texture, morphology, statistical distribution, etc., in MRI images. This involves extracting and calculating specific features within the different sequence images to assist in enhancing the predictive performance of the model.
4. By amalgamating insights derived from experimental results of Radiomics features, imaging characteristics of MRI sequences, and knowledge of neuroradiological diagnosis, we manually create four Diag-Features, with a focal emphasis on the Enhancing Tumor (ET), Necrotic and Non-Enhancing Tumor(NCR/NET) and ED(Edema). regions of brain gliomas. These features further enhance the model’s performance.

The first chapter of this paper introduces the background and innovative aspects of our work. In the second chapter, we present relevant works on glioma staging and the concept of knowledge embedding, while also discussing the role played by knowledge rules in feature extraction. The third chapter introduces the materials and methods used in this study. Our experimental results of the proposed modules are presented in the forth chapter. In the fifth chapter, the roles of each module in the paper are discussed. Finally, the paper concludes with a summary of the entire work in the sixth chapter.

## Related works

The features used for brain glioma staging tasks are primarily divided into two categories: (1) Deep features refer to the design of Convolutional Neural Network (CNN)-based feature extraction networks to extract features from images, thereby improving classification performance through enhanced classifiers and training strategies. Deep features can be extracted end-to-end from raw images without the need for manually designing feature extractors. Highly abstract and semantically rich feature representations can effectively capture both local and global information in the images. Rehman et al. [15] designed a three- dimensional CNN architecture for extracting deep features from brain tumors, followed by feature selection and classification using a feedforward neural network; Narmatha et al. [16] combined fuzzy optimization and brainstorm optimization techniques for glioma classification tasks, iteratively optimizing the network structure through fuzzy execution; Sharif et al. [17] employed a hybrid segmentation histogram equalization and ant colony optimization method to enhance image contrast. This enriched data was then fed into a meticulously designed nine-layer CNN model for deep feature extraction. Subsequently, feature concatenation and optimization via differential evolution and firefly optimization were performed before training with multi-class support vector machines (MC-SVM); Deepa et al. [18] used CNN for feature extraction and used the extracted features as inputs for deep residual networks (DRN) for classification. The networks were trained using the combination of the Jaya algorithm, honey badger algorithm (HBA), and an integrated algorithm with a time concept (CJHBA) to achieve better classification performance; Bidkar et al. [19] proposed a deep belief network (DBN) based on Salp Water optimization for brain tumor classification, mining features using CNN, and then utilizing SWO-DBN technology to effectively classify brain tumors based on the extracted features. Although improvements in classifiers or training strategy have enhanced the overall model performance, the inherent challenge of small sample data has not been fundamentally resolved in terms of the quality of feature acquisition. (2) Manual features typically require experts to design feature calculation methods based on specific medical knowledge and task requirements, including statistical, morphological, and textural aspects (such as LBP, GLCM, etc.), for manual extraction. Each feature has its physical significance, thus manual features often exhibit good interpretability. These features are often combined with machine learning algorithms such as Support Vector Machine (SVM), Random Forest, etc. Jemimma et al. [20] used fractional probability fuzzy C-means (FRF-PFCM) for automated segmentation of brain glioma MRI data, followed by feature extraction using descriptors, empirical mode decomposition (EMD), local direction pattern (LDP), wavelet transform, etc. The resultant feature set was then input into a deep belief network based on whale cat swarm optimization (WCSO-DBN) for classification. Suárez-García et al. [21] extracted texture features from MRI sequences of brain gliomas and proposed an undersampling method to divide the data into different training subsets for extracting complementary information. They combined multiple linear regression (MLR) to create different classification models. Those methods are a form of feature engineering and, while the extracted features are more precise, they still need to improve the breadth (quantity) of features for small-sample glioma image data. To address the challenges of small-sample glioma imaging data, previous literature has mainly relied on techniques from computer vision, such as data augmentation, simplifying model complexity, expanding the sample space through generative techniques, and improving classifier algorithms. However, from the perspective of external information acquisition, these methods primarily focus on the optimizing target task within the given dataset and do not introduce new external information into the model.

Therefore, the key to addressing the problem of small-sample is how to extract higher-quality features from a limited dataset [22]. To achieve this beyond the given dataset, embedding knowledge characteristics to impose reasonable rules and constraints can help the model to better understand and utilize small-sample data. These methods can be broadly categorized into two directions [23]:

The first direction is embedding knowledge information applicable to wide range of basic image types and disease diagnoses—referred to as high-level knowledge. This encompasses strategies such as: (1) transferring feature extraction models from large-scale natural image datasets or other medical images: Wang et al. [24] utilized homologous breast cancer data and transfer learning methods to train a CNN structure. They obtained Deep Learning Features (DLF) by fine-tuning the fully connected layers of the CNN structure. Using Support Vector Machines (SVM), they constructed Deep Learning Radiomics Features (DLRS) and integrated clinical factors with DLRS, proposing a LN transfer status classifier. Saini et al. [25] introduced a novel deep neural network architecture based on VGG16. They transferred domain knowledge from a larger ImageNet object dataset to a smaller and imbalanced breast cancer dataset. (2) Emulating the training routines followed by radiologists by gradually increasing task difficulty through methods like Curriculum Learning (CL): Gracias et al. [26] applied the Self-paced Curriculum Learning strategy to 3D-CNN as a method that combines medical knowledge to enhance the performance of early Alzheimer’s disease diagnosis networks. Yang et al. [27] proposed severity-guided multiple instance curriculum learning, a curriculum that progresses from easy to difficult, utilizing image training models for the classification of pathological images. (3) Using attention mechanisms and other methods to mimic the specific regions or sequences that doctors focus on. Xie et al. [28] proposed a strategy called Domain Guided-CNN, aiming to incorporate edge information in breast ultrasound (BUS) images for cancer diagnosis tasks to mimic doctors’ diagnostic behavior. Zhou et al. [29] proposed a method called Non-Local Fourier Attention, which combines self-attention mechanism with Fourier transform to capture distant spatial dependencies in the frequency domain of MRI. Xie et al. [30] proposed a Multi-scale Efficient Network (MEN), which integrates various attention mechanisms to achieve comprehensive extraction of detailed features and semantic information in COVID-19 images through progressive learning.

The other direction is embedding knowledge information applicable to particular images or specific disease diagnoses—referred to as low-level knowledge. This involves strategies such as: (1) Incorporating special features that medical professionals focus on in certain disease diagnoses (including manually created features). These features are then integrated into the network as specific patches, a process known as feature-level fusion. Alternatively, distinct classifiers are individually trained and later weighted for fusion at the decision level, known as decision-level fusion [31]. Bogacsovics et al. [32] created lesion-related hand-crafted features from fundus images and combined them with deep features for diabetes classification tasks. Ranjbarzadeh et al. [33] proposed various encoding methods beneficial for detecting target textures to encode breast cancer X-ray images to eliminate the pectoral muscle portion. They then used local patches as input and concatenated all extracted features into a vertical vector to apply to fully connected layers for pixel-wise classification, achieving promising results. (2) Leveraging specific image feature calculation methods that focusing on texture, morphology, grayscale distribution, and other aspects to more effectively extract features to enhance the model’s prediction capabilities. Xiao et al. [34] extracted radiomic features from brain tumor MRI scans from 9 different regions of interest (ROI). They utilized LASSO and Principal Component Analysis (PCA) for feature selection and finally trained and predicted using Logistic Regression and Random Forest models to detect meningioma patients, achieving satisfactory results. Al-Mekhlafi et al. [35] combined deep features extracted from DenseNet-121 and ResNet-50 with handcrafted features such as GLCM, FCH, and DWT for the diagnosis of malignant lymphoma. Bagherian Kasgari et al. [36] proposed a robust and efficient brain tumor segmentation pipeline based on machine learning. Initially, they utilized the Zernike moment method to extract multiple feature maps from MRI, thereby generating different sets of edge feature maps. Simultaneously, they employed Local Directional Number Pattern (LDNP) for feature extraction. Finally, Enhanced Ant Lion Optimizer (EALO) was used to find the optimal feature map set for creating the best encoded images. Additionally, various imaging techniques exhibit differences in sensitivity to biological structures and lesions, spatial resolution, and the ability to capture tissue properties. This variability leads to unique focus areas, necessitating the consideration of differences among imaging technologies to ensure accurate diagnosis and analysis. In our survey, we found that there is limited research considering this factor, making it challenging to form effective references.

## Materials and methods

**Fig 1:**
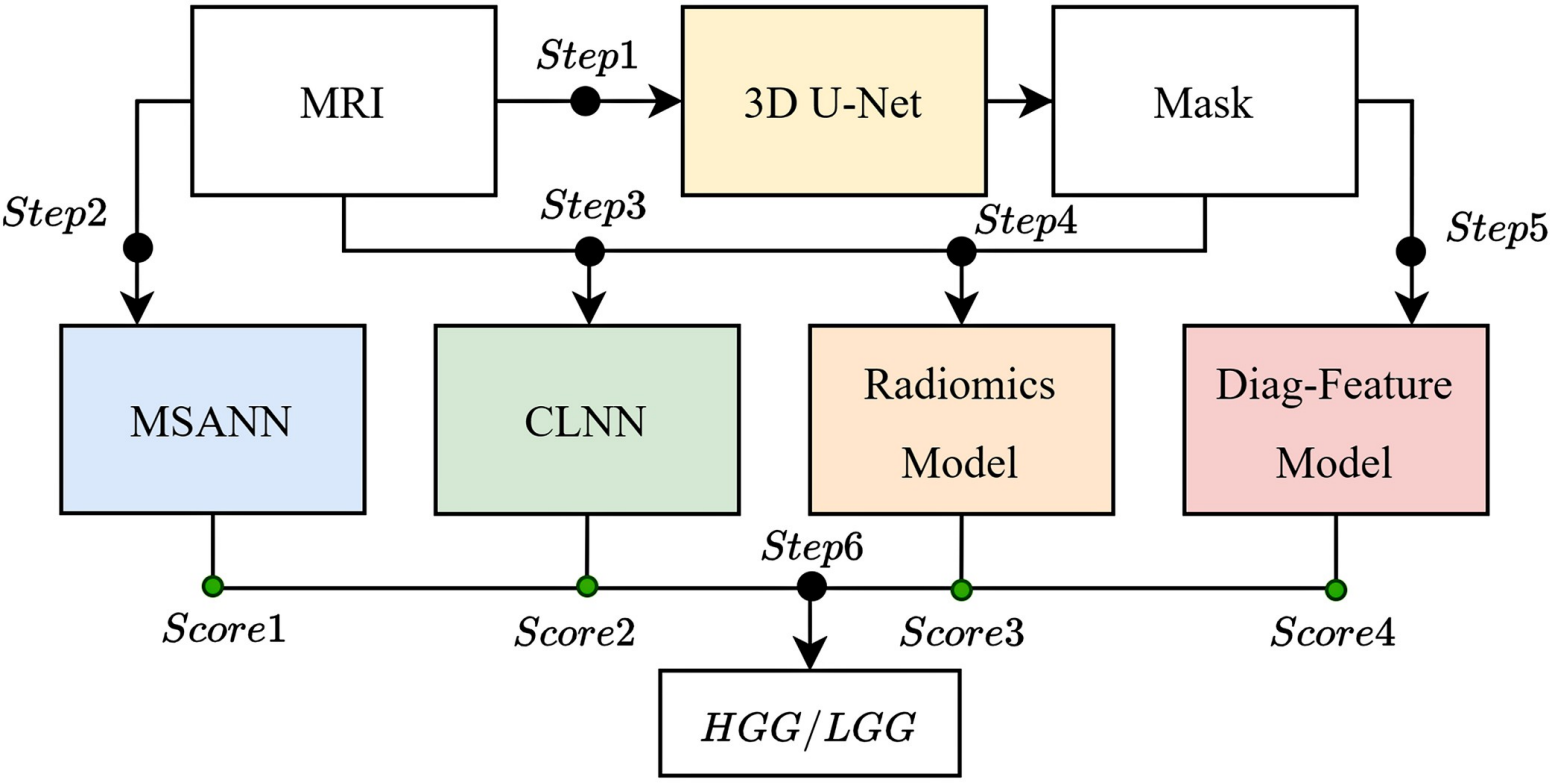
The flowchart of the MCE model.

In this study, we propose an approach to embed knowledge characteristics into the feature extraction stage, incorporating the learning process of radiologists during image interpretation, adiomics, the specificity of different MRI sequences [37, 38] (Table 1), and key points in neuroradiological diagnosis of brain gliomas into the model design. This is employed for the automatic classification of (GBM and non-GBM using patient MRI images. Considering that optimal tumor image slices may not always be guaranteed, our overall model strategy is based on 3D data design to better adapt to practical applications in tumor image analysis. The overall design of the model is as Fig 1:

Step 1: Use 3D U-Net to segment the images and obtain masks for three lesion regions (NCR/NET, ET, ED).
Step 2: After image preprocessing, input them into MSANN for training and calculate Score 1.
Step 3: Input the MRI and its corresponding segmentation mask into CLNN for training and calculate Score 2.
Step 4: Input the MRI and its corresponding segmentation mask into the Radiomics model for training and calculate Score 3.
Step 5: Input the segmentation mask into the Diag-Feature for training and calculate Score 4.
Step 6: Obtain the final result by averaging the scores obtained above through voting.

**Table 1:** Function of MRI different sequence imaging.

Sequence	Function
FLAIR	FLAIR enhances the display of edema areas around tumors by suppressing fluid signals, determining the boundaries of the tumor.
T1CE	T1CE, after the injection of contrast agent, can more clearly depict the tumor portion with increased vascular density, used to determine the tumor’s blood supply and activity.
T2	T2 can clearly reveal the tumor’s water content, boundaries, and the surrounding edema, used for determining the tumor’s type and activity.

### Data description and preprocessing

**Fig 2:**
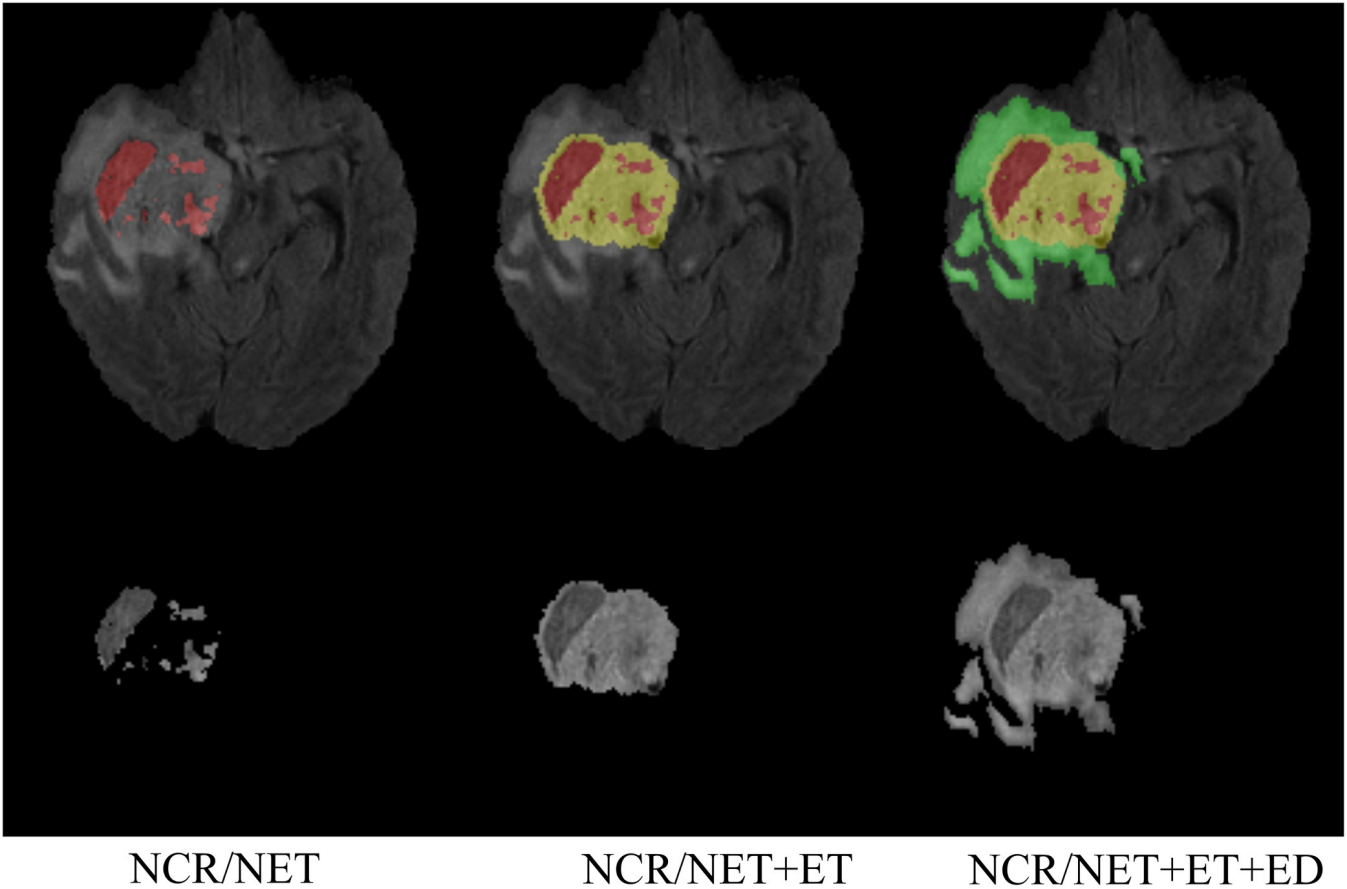
Segmentation labels for MRI images of brain gliomas. The first column composed of NCR/NET(red); the second column composed of NCR/NET(red) and ET(yellow); the third column composed of NCR/NET(red), ET(yellow) and ED(green).

**Fig 3:**
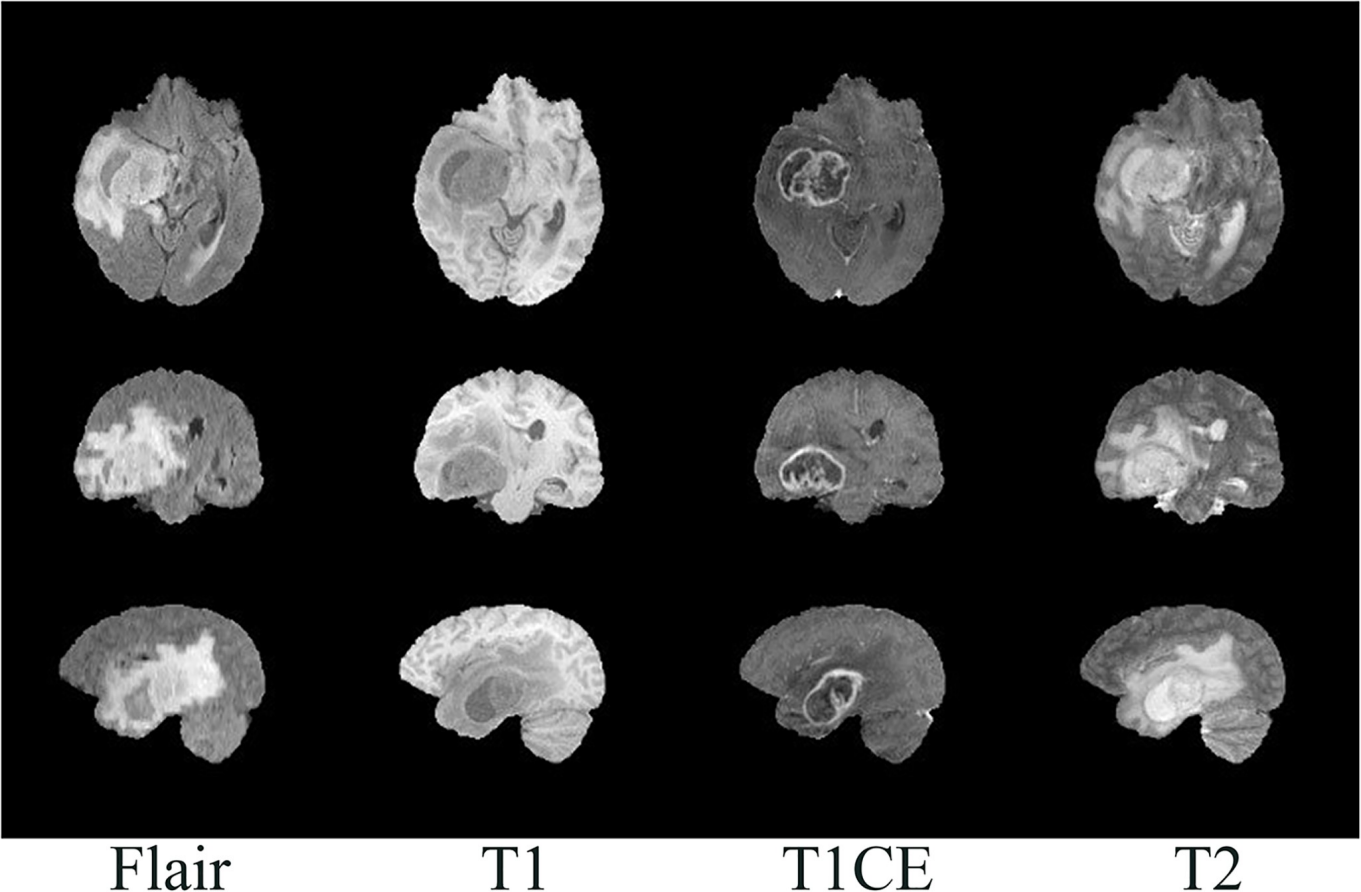
MRI images in 4 sequences and 3 sections. The first column represents the flair; the second column represents the T1; the third column represents the T1CE; the fourth column represents the T2, the first row represents the transverse plane; the second row represents the coronal plane; the third row represents the sagittal plane.

The experiments in this paper utilized two publicly available datasets from MICCAI, namely BraTS2018 (HGG = 210, LGG = 75) and BraTS2020 (HGG = 293, LGG = 76), the dataset can be obtained from http://www.braintumorsegmentation.org. It is worth noting that, unlike WHO grading, the BraTS dataset defines GBM as HGG, while I, II, III are LGG. Therefore, the essential task of this study is to differentiate between GBM and non-GBM. The dataset comprises three-dimensional MR images of brain gliomas (as shown in Figs 2 and 3), with each sample containing four MRI sequences: Flair, T1, T2, and T1ce. All images are co-registered to the same anatomical template after skull stripping. Additionally, the segmentation labels of the images are manually delineated by multiple evaluators, using MRI with the same resolution interpolation. The segmented labels include regions for ET, ED, and NCR/NET [39–41].

Due to the BraTS dataset being collected from imaging across multiple medical centers, the voxel values have different scales, with a maximum range of 0-32767 and a minimum range of 0-111. To better adapt to subsequent processing, the images were subjected to Z-score normalization, and the original size of the 240 × 240 × 155 3D images was cropped to a resolution of 160 × 160 × 160.

### Segmentation of MRI

To obtain spatial information of lesion areas and generate masks for subsequent feature extraction modules, a segmentation network based on 3D U-Net is used to segment the samples. 3D U-net is an extension of the traditional U-net [42]. Compared to other 2D-designed segmentation networks, it achieves a more balanced leading position in terms of segmentation accuracy, parameter count, and training time for the segmentation of three regions: Enhancing Tumor (ET), Tumor Core (TC, NCR/NET+ET), and Whole Tumor (WT, NCR/NET+ET+ED) [43]. 3D U-Net consists two fundamental components: an encoder and a decoder. The encoder gradually extracts feature representations from low- level to high-level, while the decoder reconstructs and fuses feature maps through upsampling and skip connections. Preprocessed MRI images are used as input, and traverse through a series of convolutional and pooling layers in both the encoder and decoder, progressively extract high-level feature representations. In the decoder part, upsampling operations gradually reconstruct feature maps, and skip connections are introduced to merge feature maps from the encoder and decoder stages, combining feature information from different levels. Finally, a 1 × 1 convolutional layer and a sigmoid activation function are applied to map the feature maps to binary segmentation masks. The generated segmentation masks not only provide spatial information for creating diagnostic features of brain gliomas but can also be used for subsequent CLNN extraction modules and radiomic feature extraction modules.

### Simulating the image interpretation process of radiologists

Radiologists, when reviewing a patient’s MRI, typically concentrate their attention on characteristic regions of different sequences. Different MRI sequences provide diverse information about tissue structure, blood flow, metabolism, and function. These regions carry richer information during the disease diagnosis process compared to others (Table 1). The paper designs two methods, respectively, at the model and strategy levels to mimic the real process of doctors interpreting medical images. The former method utilizes a CNN network structure based on attention mechanisms, while the latter achieves it through the design of curriculum learning strategies.

#### Multi- Sequence Attention Neural Network

**Fig 4:**
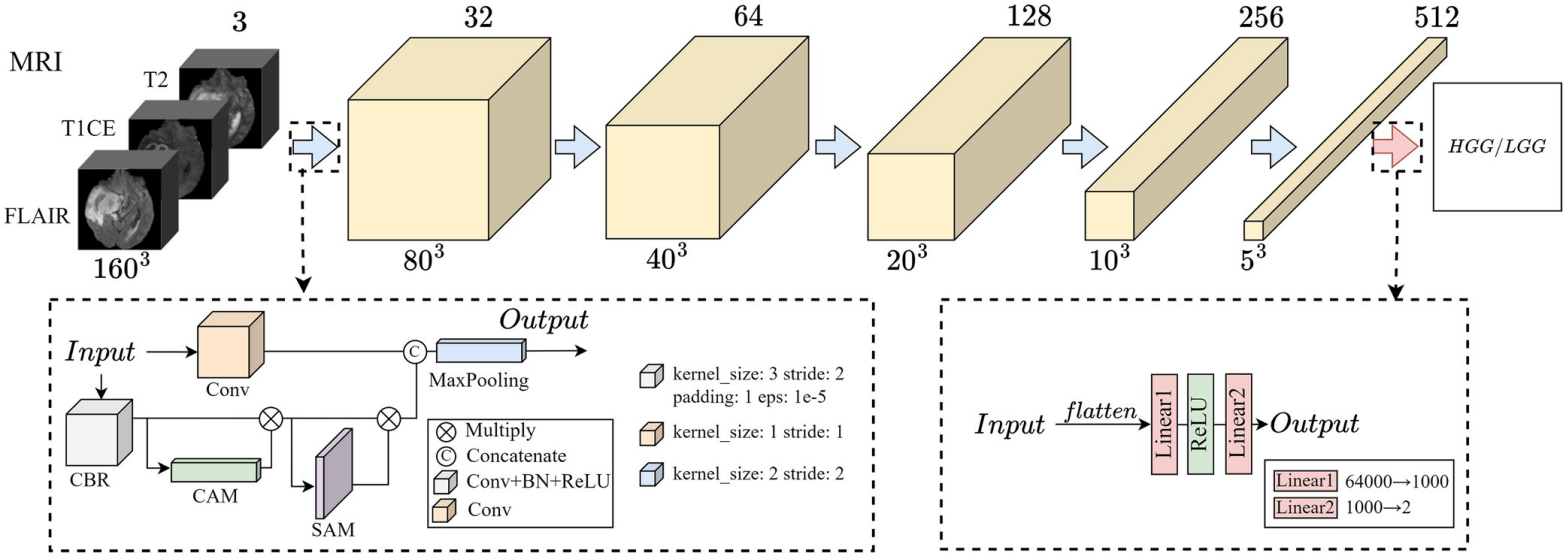
Multi- Sequence Attention Neural Network.

Doctors typically do not analyze information from all images at once when reviewing medical images. Instead, they enhance their focus on specific regions in specific sequence MRI scans. Attention mechanisms are considered a means for humans to quickly select the most important information from a large amount of data using limited processing resources [44]. This aligns well with the goal we aim to achieve. Fig 4 illustrates the model using the Multi-Sequence Attention (MSA) mechanism to mimic the attention pattern of radiologists during image interpretation for deep feature extraction. As mentioned earlier, MRI images from different sequences reflect distinct tissue information and the differences between sequences need to be carefully considered. In our model input, we take MRI data from FLAIR, T1CE, and T2 sequences as three channels and feed them into the model. We utilize the Channel Attention Module (CAM) to learn the importance of each channel (sequence), enabling the model to focus more on channels that contribute most significantly to tumor classification. To ensure that the model pay attention to lesion areas within channels that provide more information for tumor classification, while also mitigating the impact of factors such as artifacts and vignetting, we introduce the Spatial Attention Module (SAM). Initially, convolutional and pooling layers are applied to each channel to extract low-level feature representations. We use adaptive max-pooling and average-pooling to generate a global description of the features. Subsequently, these descriptions are mapped to an attention weight vector through a fully connected layer. This vector reflects the importance of each spatial location and is used to weight the feature maps, giving higher attention to lesion areas.

The output and the convolution result of the initial input are concatenated as the input of the next layer. The result is then fused with the input convolution output and passed to the next layer. This process is designed to enable the model to focus on global information, which may include aspects such as tumor invasion and compression of normal tissues. The mapping between layers is accomplished using the module outlined in the left dashed box in Fig 4. The computation formula (1) is as follows:

$$F=Conv\left(f_{x}\right)\left|\right|CAM\left(Conv\left(f_{x}\right)\right)⊙SAM\left(CAM\left(Conv\left(f_{x}\right)\right)\right)$$ (1)

where *f*_*x*_ represents the input features, *Conv* denotes convolutional computation, *CAM* stands for channel attention weighting, and *SAM* represents spatial attention weighting, || represents the concatenation operation, ⊙ represents the dot product of two vectors. Finally, the features extracted from the linear layer are input into the classifier for training, obtaining Score 1.

#### Clinical Learning Neural Network

**Fig 5:**
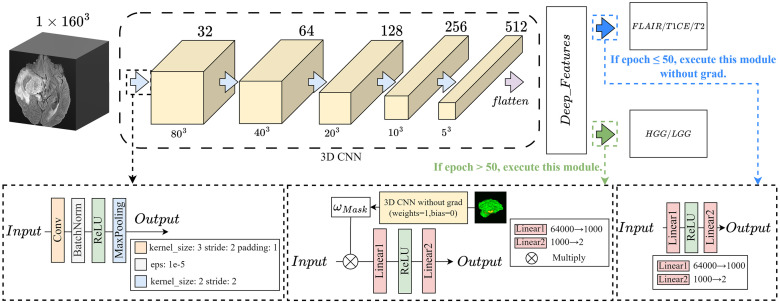
Clinical Learning Neural Network.

Curriculum Learning is a strategy used to train models with a specific curriculum. The core idea is to design an effective curriculum that mimics the human learning process, typically progressing from easy to difficult. According to the complexity of tasks during radiologists’ image interpretation, the curriculum is set—designing a CL strategy consisting of two training rounds(Fig 5): in the first round of training (Epoch ≤ 50),the model is trained with the three MRI sequences (FLAIR/T1CE/T2) as learning labels. The model is trained to differentiate between the types of input image sequences, enabling it to focus on features relevant to the image sequences. In the second round(Epoch >50), the feature extraction model trained in the first round is transferred to the brain glioma classification task (GBM/non-GBM). Additionally, in this round, the weights *ω*_*Mask*_, corresponding to the masks of relevant lesion areas, are multiplied in the network. This gradually directs the model’s attention towards the lesion areas. By setting a pre-task, the distinct imaging characteristics of MRI sequences and information from characteristic regions can participate in the glioma staging, making the learned features more reasonable and effectively alleviating overfitting issues in the model.

Acquiring *ω*_*Mask*_: Input the segmentation results of U-Net into a 3D CNN with initialized weights (weights = 1, bias = 0), record the flattened one-dimensional vector as the mask. Set weights for the mask: on FLAIR, the weights for ED and ET regions are set to 1.5, while other regions are set to 1; on T1CE, the weights for ET and NCR/NET regions are 1.5, while other regions are 1; on T2, the weights for NCR/NET, ET, and ED regions are 1.5, while other regions are 1. Finally, input the weighted features into the classifier for training and computation, obtaining Score 2.

### Radiomics

In this study, Radiomics methods are employed to extract features from brain glioma images. In medical imaging, there may be feature information that reflects lesions but goes beyond visual interpretation. Fully utilizing this information may enable the macroscopic decoding of the structural phenotypes of many lesions. Radiomics is a technique for quantitatively extracting features from medical images that can extract information such as texture, morphology, and grayscale distribution within images [45]. This information is then converted into numerical features. thereby constructing high-dimensional feature vectors for the analysis of brain glioma MRI.

**Fig 6:**
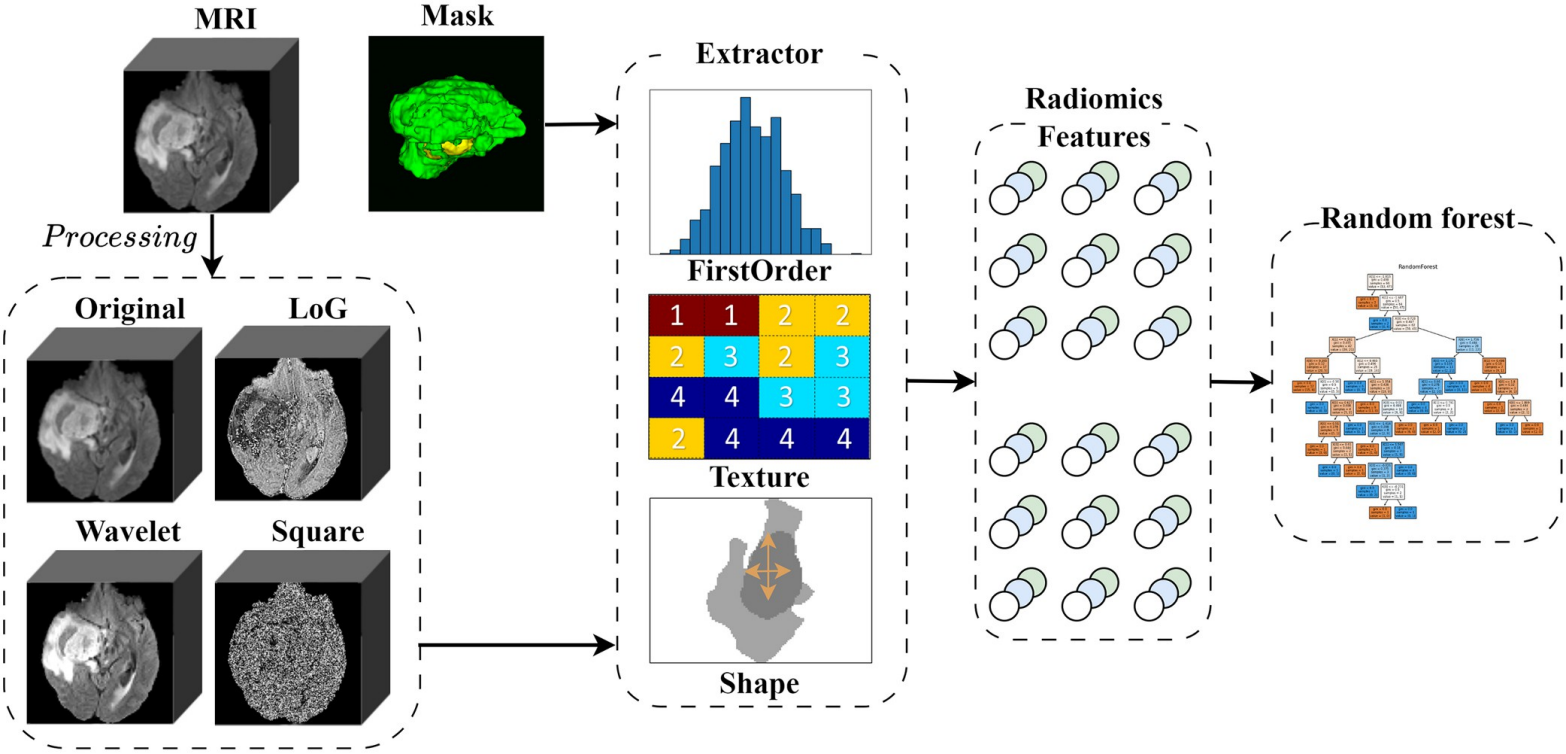
Radiomics is used for feature extraction.

Fig 6 illustrates the Radiomics model. Firstly, the original images are transformed using methods such as LoG, Wavelet, and Squared. Next, the segmentation mask is applied to both the transformed and original images to eliminate the background and retain different lesion areas related to the tumor. Subsequently, a series of feature extraction methods are employed for feature extraction and computation, such as Gray-Level Co-occurrence Matrix (GLCM), gray-level statistics, shape descriptors, etc. This includes specific aspects as follows [46]:

- Texture features, which include energy, entropy, contrast, and correlation, are used to describe the texture characteristics of brain tumor images;
- Morphological features, including volume, surface area, maximum diameter, and perimeter, are employed to describe the morphological characteristics of brain tumors;
- TGray-level statistical features, comprising mean, standard deviation, kurtosis, and skewness, are used to describe the grayscale distribution characteristics of brain tumors.

Finally, random forest classifiers were established for a total of six combinations, targeting two types of masks (TC and WT) and three sequences, to conduct training and computation for obtaining Score 3. It is important to note that the scores for each combination directly contribute to the weighted calculation of the final score 3.

### Brain glioma diagnostic rules and diagnostic features

In brain glioma MRI diagnosis, grades I, II, and III of brain gliomas typically exhibit high signal intensity on T2 and FLAIR with relatively limited enhancement areas. In contrast, the GBM exhibit more extensive ET regions in the images, often accompanied by significant ED regions in the peripheral ET regions, and the central region may show distinct necrotic features–NCR/NET regions. Additionally, in the subsequent prediction results of Radiomics features, the features extracted from the T1CE show more prominent performance. This provides important guidance for feature selection. Taking into account the distinct characteristics of the T1CE sequence, we believe that the ET region may be more crucial for glioma classification. Based on the above rules, we consider the ratio of ET to ET+ED volume and the ratio of ET to NCR/NET+ET volume may be crucial for distinguishing GBM. We further transform these observed results into diagnostic features (Diag-Feature) *F*_3_ and *F*_4_. Additionally, to reduce linear redundancy among features, *F*_3_ and *F*_4_ are respectively subjected to nonlinear processing to obtain *F*_1_ and *F*_2_. The specific calculation formulas are as follows:

$$F_{1}=ln\left(V_{ET}+N\right)-ln(V_{ET}+\left(V_{NCR/NET}+N\right),$$ (2)

$$F_{2}=ln\left(V_{ET}+N\right)-ln(V_{ET}+\left(V_{ED}+N\right),$$ (3)

$$F_{3}=\frac{V_{ET}+N}{V_{ET}+V_{NCR/NET}+N},$$ (4)

$$F_{4}=\frac{V_{ET}+N}{V_{ET}+V_{ED}+N},$$ (5)

where *V*_*ET*_ represents the volume of the ET region, *V*_*NCR*/*NET*_ represents the volume of the NCR/NET region, *V*_*ED*_ denotes the volume of the ED region, and *N* is a constant. We conducted T-test and Pearson Correlation Coefficient analyses on the four formulated features to ensure that the statistical correlation between these features and the labels is significant. Finally, we established a Logistic Regression model to train and predict Diag-Features, obtaining Score 4.

### Prevent overfitting

Acquiring and annotating medical imaging data is often costly and time-consuming, resulting in relatively limited available data. Scarcity of data may lead to insufficient coverage of certain classes or features in the training set. This can prevent the model from capturing the true distribution of the data during the learning process, leading to the memorization of features from only a few samples. Additionally, extracted features typically have high dimensionality, requiring the model to learn complex features to distinguish between different pathological conditions. A lack of data can cause the model to overfit to noise or local features in the training data while ignoring the overall data distribution. In such cases, the model is prone to overfitting due to insufficient samples.

The proposed method aims to reduce overfitting from two perspectives: (1) From the overall perspective of the scheme, we employ a combination of four models to overcome the problem of model overfitting. At the design stage of the overall scheme, we intentionally allow different models to capture and learn different patterns and modes in the data. This diversity makes them prone to errors in certain situations but correct in others. When their predictions are combined, errors can be mutually offset, while the correct parts are retained, effectively reducing the random fluctuations caused by the high complexity of a single model. (2) From the perspective of individual modules, in the 3D U-Net, MSANN, and CLNN modules, we primarily mitigate model overfitting by reducing network depth and incorporating normalization layers and Dropout layers. In the Radiomics module, we use random forests for feature selection and modeling, primarily overcoming model overfitting by adjusting the number of decision trees in the random forest. In the Diag-Feature Model module, as the number of features used is small, we did not encounter severe overfitting issues.

## Results

### Implementation details

Training was conducted concurrently on two RTX 4090 GPUs, an Intel Xeon(R) Silver 4110 CPU @ 2.10GHz, and 32GB of RAM, utilizing the PyTorch framework. The proposed method was tested on the BraTS2018 and BraTS2020 datasets. We split the dataset into 80% for training and 20% for test. We used Ry Radiomics V3.0.1 [47] to design the Radiomics features extraction module, as configured in Table 2 of the PyRadiomics settings. If feasible in the future, our code will be made available as open source on https://github.com/Turing17/MCE_model.

**Table 2:** Parameter setting in PyRadiomics.

Parameter name	Value
ImageType	Original, LoG, Wavelet, Square;
FeatureClass	glcm, glrlm, firstorder, shape2D, glszm, ngtdm, gldm, shape;
Setting	normalize: True, binWidth: 25.

All the experiments underwent 5 rounds of cross-validation. In the method, the hyperparameters for training MSANN were set as follows: the learning rate was 5 × 10^−4^, Epochs set to 30 when using BraTS2020 and 25 when using BraTS2018, Batch size was 2, loss parameter was CrossEntropyLoss, and optimizer was Adam. For training CLNN, the hyperparameters were set to a learning rate of 5 × 10^−4^, Epochs of 180, Batch size of 2, CrossEntropyLoss as the loss parameter, and Adam as the optimizer. In the 3D U-Net segmentation module, the hyperparameters are set as follows: the learning rate is 15 × 10^−4^, Epochs is 100, the optimizer is Adam, and the Batch size is 2. and the loss was defined as per Formula 6:

$$L_{DiceBRD}=1-\frac{2\sum_{i=1}^{voxels}\sum_{c=0}^{C}y_{ic}p_{ic}+\varepsilon }{\sum_{i=1}^{voxels}\sum_{c=0}^{C}\left(y_{ic}+p_{ic}\right)+\varepsilon }-\frac{1}{N}\sum_{i=1}^{N}\sum_{c=0}^{C}y_{ic}log\left(p_{ic}\right),$$ (6)

where *voxels* represents the total number of voxels, ∈ is a very small number; *y*_*ic*_ ∈ [0, 1] represents the one-hot encoding for voxel *i* belonging to class *c*; *p*_*ic*_ ∈ [0, 1] is the probability that voxel *i* is predicted as class *c*; *N* is the total number of voxels, and *C* is the number of classes (including the background).

Regarding the hyperparameter tuning of 3DU-Net, MSANN, and CLNN, the main objective is to use Dice and Accuracy as the target metrics, and adjust parameters such as learning rate, batch size, Dropout ratio, optimizer, etc., based on relevant parameter tuning experience. In the Radiomics module and the Diag-Feature Model module, grid search is primarily used to find the best hyperparameter combinations. These two types of adjustments are independent but both use Accuracy as the determining criterion.

The curves used in the experimental results figures have all undergone smoothing.

### Evaluation index

To evaluate the performance of the proposed method, this paper adopts the commonly used Dice Similarity Coefficient (DSC) to assess the segmentation performance of the 3D U-Net:

$$DSC=\frac{2TP}{2TP+FP+FN}$$ (7)

Using Accuracy, Recall, and Precision to evaluate the classification performance of each model for GBM and non-GBM:

$$Accuracy=\frac{TP+TN}{TP+TN+FP+FN}$$ (8)

$$Recall=\frac{TP}{TP+FN}$$ (9)

$$Precision=\frac{TP}{TP+FP}$$ (10)

where *TP* represents the model’s correct prediction of positive class results. *TN* represents the model’s correct prediction of negative class results. *FP* represents the model’s incorrect prediction of negative class as positive results. *FN* represents the model’s incorrect prediction of positive class as negative results.

### Segmentation

In the segmentation network based on the 3D U-Net, we reduced the network’s depth to mitigate overfitting in situations with limited data and to save training costs. We combined the MRI sequences of FLAIR, T1CE, and T2 into a tensor of size 3 × 160 × 160 × 160 and input it into the model for training. To facilitate efficient training, we further divided the input tensor into 5 smaller tensors of size 3 × 32 × 160 × 160 and used them for training. The segmentation results for each region are presented in Table 3.

**Table 3:** DSC of segmentation.

Dataset	ET	TC	ET	Average
BraTS2018	0.791±0.047	0.824±0.042	0.886±0.021	0.834
BraTS2020	0.795±0.035	0.831±0.039	0.893±0.027	0.840

### Multi- Sequence Attention Neural Network

**Fig 7:**
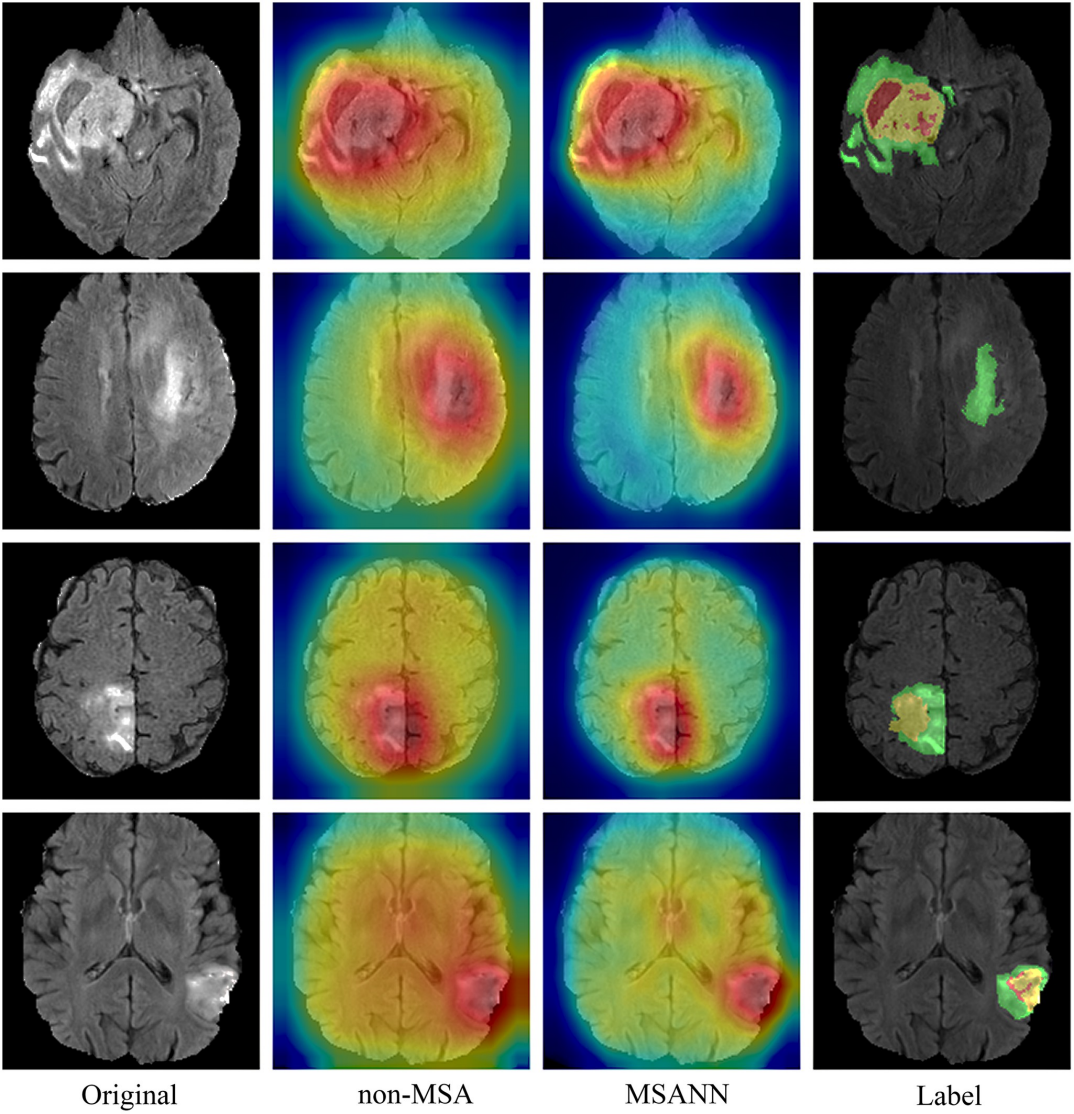
Class activation map of feature layer for feature extraction using the neural network without MSA and MSANN. The first column is original image, the second column is feature extraction using the Neural Network without MSA, the third column is feature extraction using MSANN, and the fourth column is ground truth segmentation labels.

**Fig 8:**
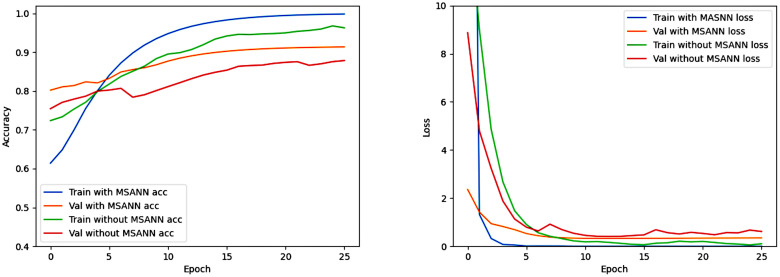
Training-validation accuracy and loss curves of MSANN and the neural network without MSA using the BraTS2018 dataset.

**Fig 9:**
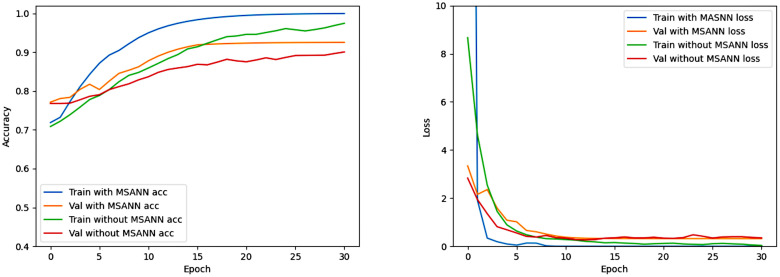
Training-validation accuracy and loss curves of MSANN and the neural network without MSA using the BraTS2020 dataset.

To validate that the designed attention mechanism module effectively focuses on lesion regions for better feature extraction, the following verifications were conducted: We used Gradient-weighted Class Activation Mapping (Grad-CAM) technique to visualize the specific image regions associated with predictions, providing an intuitive comparison of the regions the model pays attention to. From Fig 7, it can be seen that the network with the attention mechanism can more precisely focus on information-rich lesion regions, thereby effectively increasing the information density of feature extraction. As shown in Table 4, Figs 8 and 9, the comparative results of using MSANN and directly using a convolutional neural network on BraTS2018 and BraTS2020 are presented. The features extracted by MSANN contain more relevant information compared to the features extracted by the convolutional neural network without MSA mechanism, resulting in better classification accuracy.

**Table 4:** Results of MSANN and the neural network without MSA using the BraTS2018 and BraTS2020 datasets.

Method	Datasets	Accuracy(%)	Recall(%)	Precision(%)
Without MSA	BraTS2018	89.47	82.72	82.99
	BraTS2020	90.80	83.10	90.63
MSANN	BraTS2018	91.52	87.08	92.24
	BraTS2020	92.97	85.14	91.94

### Clinical Learning Neural Network

**Fig 10:**
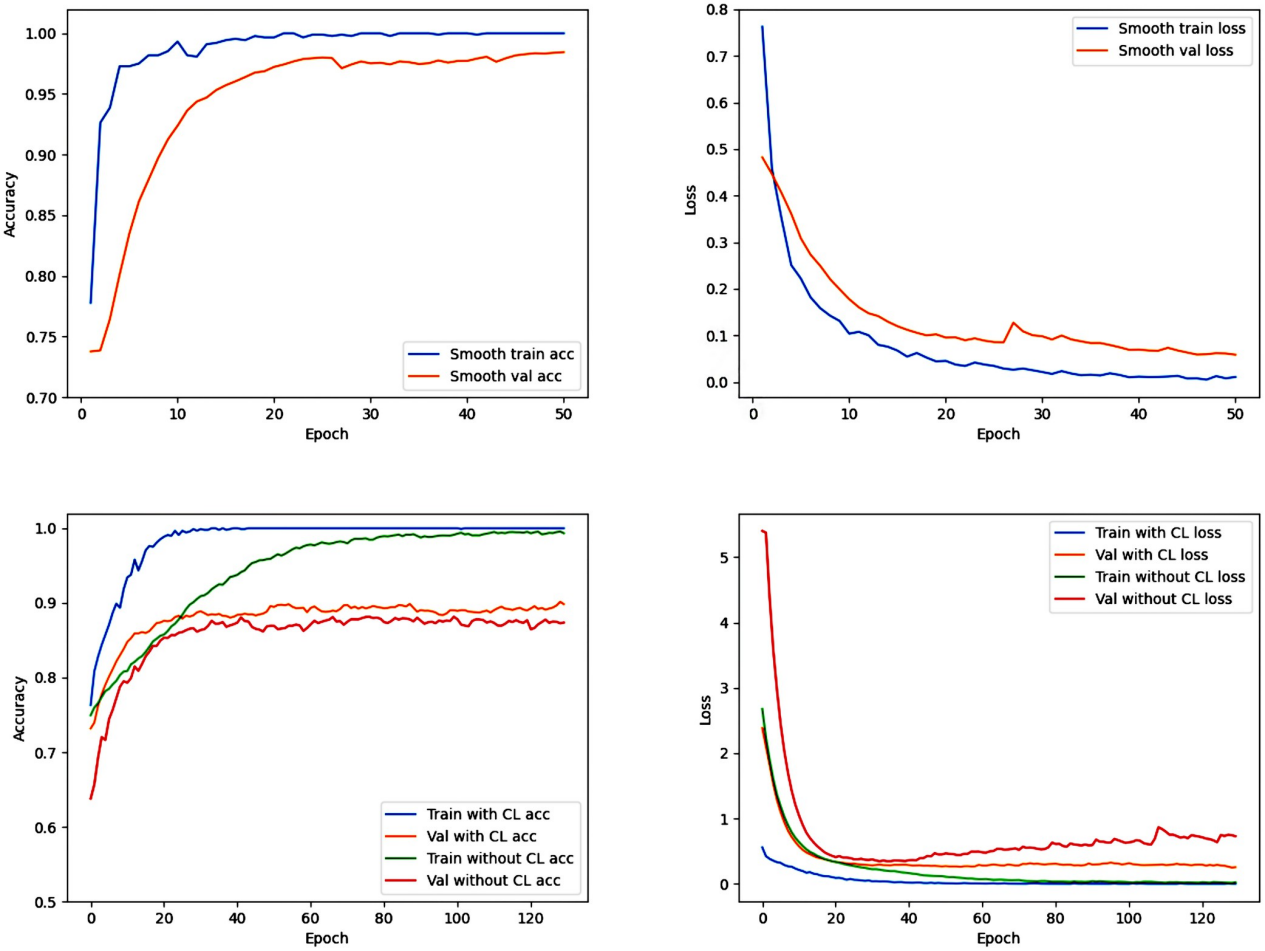
Training-validation accuracy and loss curves of CLNN and the neural network without curriculum learning using the BraTS2018 dataset. The first row of images represents the first 50 epochs (task based on image sequences), and the second row of images represents the last 130 epochs (task based on glioma grades).

**Fig 11:**
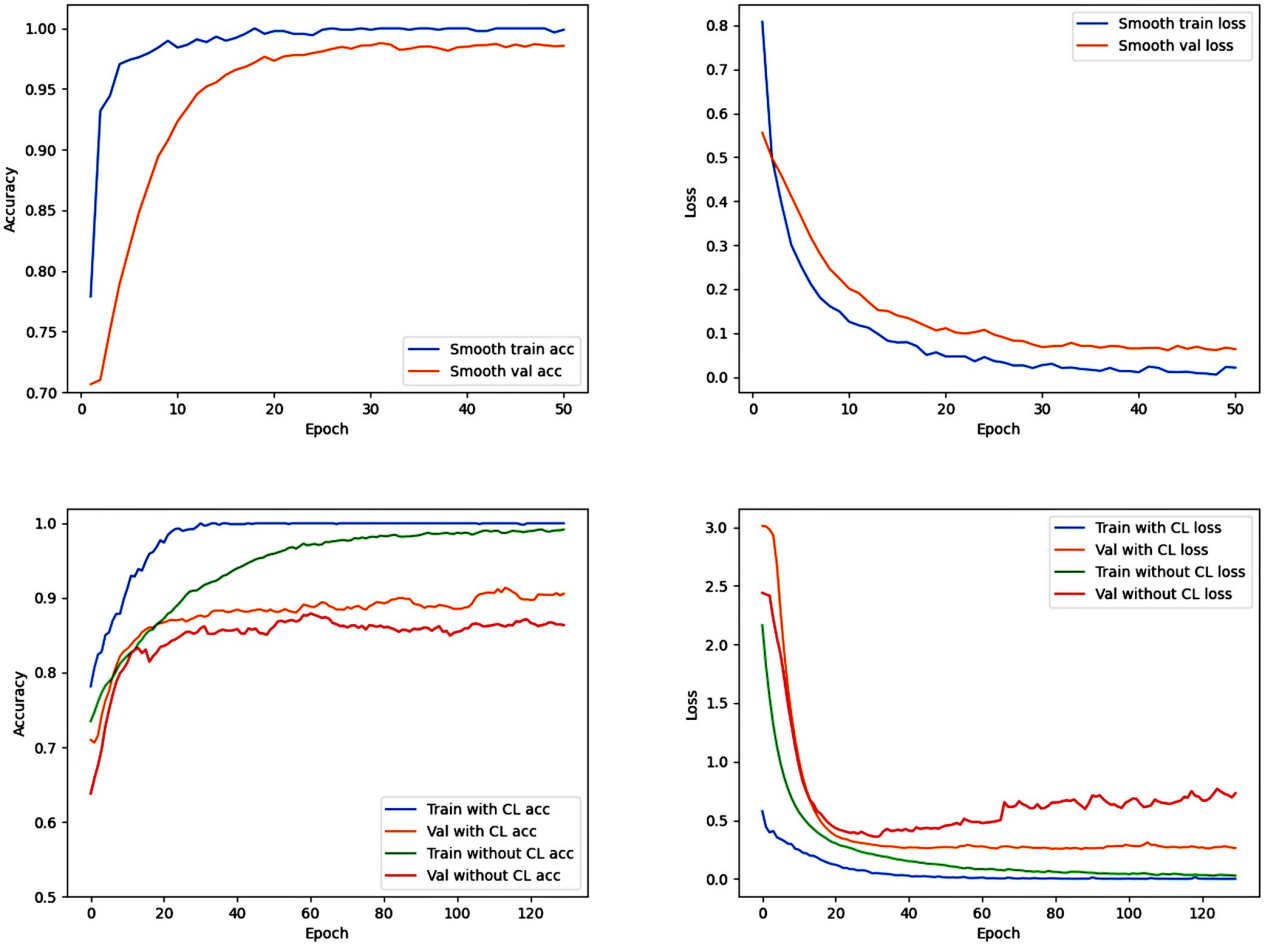
Training-validation accuracy and loss curves of CLNN and the neural network without curriculum learning using the BraTS2020 dataset. The first row of images represents the first 50 epochs (task based on image sequences), and the second row of images represents the last 130 epochs (task based on glioma grades).

CLNN is trained in the first round (Epochs ≤ 50) with the sequences of MRI images as labels, and the best model is selected to transfer to the second round (Epochs > 50) of training. The specific training situation is shown in Figs 10 and 11. In the task of classifying MRI sequences, the Accuracy using the BraTS2018 and BraTS2020 datasets is 99.56% and 99.41%, respectively. We compared the results of two methods in glioma classification tasks: one using this curriculum learning strategy and the other directly executing the model for 50 rounds. CLNN achieved Accuracy, Recall, and Precision of 92.28%, 88.41%, 93.22% on BraTS2018, and 92.70%, 87.60%, 89.36% on BraTS2020, respectively, improving by 1.4%, 2.78%, 3.76% and 1.35%, 5.10%, 3.77%, respectively(Table 5, Figs 10 and 11).

**Table 5:** Results of CLNN and neural network without curriculum learning using the BraTS2018 and BraTS2020 datasets.

Method	Datasets	Accuracy(%)	Recall(%)	Precision(%)
Without CL	BraTS2018	90.88	85.63	89.46
	BraTS2020	91.35	82.50	85.59
CLNN	BraTS2018	92.28	88.41	93.22
	BraTS2020	92.70	87.60	89.36

Through comparison, it was observed that compared to CLNN, the model without using the CL strategy exhibited severe overfitting after 40 rounds of training. At the same time, the model trained with the CL strategy was able to converge to a better solution more quickly during training. This is because, in contrast to indiscriminately inputting various MRI sequences into the network, the CL strategy can help the model better adapt to different data distributions and input conditions, enhancing the model’s tolerance to noise and variations. Additionally, this strategy encourages the model to gradually learn complex feature representations rather than merely memorizing the training data, aiding the model in better understanding the essence of the problem rather than simply memorizing patterns in the training set.

### Radiomics model

**Fig 12:**
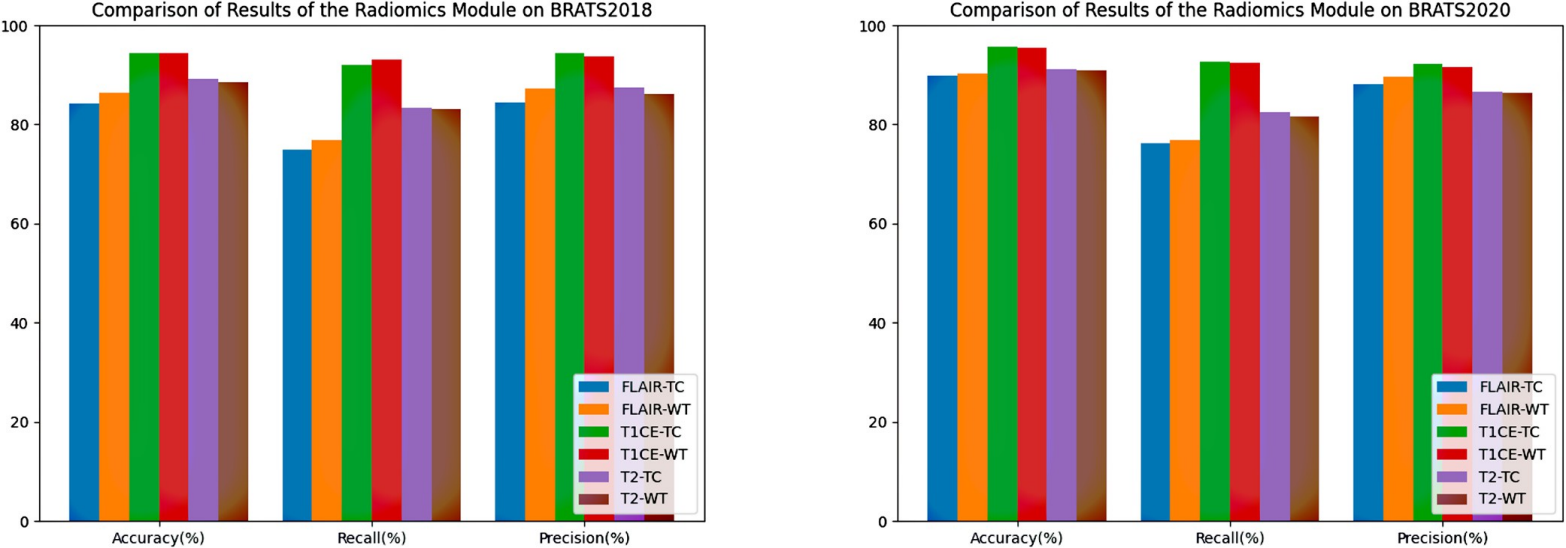
Bar chart illustrating the results of the radiomics model using the BraTS2018 and BraTS2020 dataset.

In the module for extracting Radiomics features, FLAIR, T1CE, and T2 sequences of MRI are used respectively as masking regions for TC and WT for imaging genomics feature extraction. After undergoing LoG, Wavelet, and Square transformations, each sequence obtained 1093 features, including texture features (GLCM, GLRLM, GLDM, NGTDM, GLSZM), first-order statistical features, and morphological features. Subsequently, the 6 sets of features are inputted into random forests for training and testing. In addition to using Accuracy, Recall, and Precision as evaluation metrics, we also conducted permutation tests on the predicted results of TC and WT regions to ensure that the features extracted from different regions have distinctiveness. The results are shown in Fig 12, Tables 6 and 7.

**Table 6:** Results of the six combinations using the BraTS2018 Radiomics models.

Method	Accuracy(%)	Recall(%)	Precision(%)	Permutation test P-value
FLAIR-TC	84.21	74.80	84.31	0.038
FLAIR-WT	86.32	76.91	87.19	
T1CE-TC	94.39	92.04	94.35	0.03
T1CE-WT	94.37	93.09	93.75	
T2-TC	89.12	83.33	87.54	0.049
T2-WT	88.42	83.17	86.24	

**Table 7:** Results of the six combinations using the BraTS2020 Radiomics models.

Method	Accuracy(%)	Recall(%)	Precision(%)	Permutation test P-value
FLAIR-TC	89.73	76.15	88.17	0.026
FLAIR-WT	90.27	76.88	89.61	
T1CE-TC	95.68	92.58	92.14	0.001
T1CE-WT	95.41	92.41	91.56	
T2-TC	91.08	82.39	86.59	0.190
T2-WT	88.42	83.17	86.24	

On both BraTS2018 and BraTS2020 datasets, the classification results based on features extracted from the T1CE sequence are higher than those from other sequences. This is because the imaging effect of the T1CE sequence on the ET and NCR/NET regions is superior, making it richer in information. This is crucial for the staging of gliomas, providing valuable guidance for the subsequent design of diagnostic feature computation methods. Meanwhile, it can be seen from the results of permutation tests that, except for the results on the T2 sequence of BraTS2020, the P-values of the other items are all less than 0.05, indicating significant differences. This indicates that selecting two regions for radiomics feature extraction in the experiment indeed contributes significantly to the prediction of GBM, without the occurrence of feature redundancy.

### Diag-Feature model

To validate the relationship between the selected manual Diag-Feature and glioma staging, we conducted independent Pearson Correlation Coefficient and T-test analyses to assess the correlation between these four diagnostic features and the results. Pearson Correlation Coefficient was used to evaluate the correlation between each of the four diagnosis features and the results, as shown in Table 8. The results confirm that all four manual diagnostic features have P-values from the T-test calculations that are below 0.05, indicating significant statistical significance.

**Table 8:** Results of Precision CC and T-test for the four manually crafted features.

Feature	Dataset	Precision CC	T-test	
			t-statistic	p-value
*F*_1_	BraTS2018	0.6630	14.9000	1.8323 × 10^−37^
	BraTS2020	0.6719	17.3810	8.49703 × 10^−50^
*F*_2_	BraTS2018	0.6200	13.2925	1.1743 × 10^−31^
	BraTS2020	0.6270	15.4180	1.0519 × 10^−41^
*F*_3_	BraTS2018	0.6888	15.9821	2.0375 × 10^−41^
	BraTS2020	0.6682	17.2045	4.5918 × 10^−49^
*F*_4_	BraTS2018	0.4295	8.0019	3.1762 × 10^−14^
	BraTS2020	0.3941	8.2135	3.7112 × 10^−15^

From the Pearson Correlation Coefficient and T-test results, it can be observed that features *F*_1_,*F*_2_ and *F*_3_ exhibit a high degree of correlation with glioma staging, while feature *F*_4_ demonstrates a moderate level of correlation. Table 9 displays the results of training the manual diagnostic features on a Logistic regression model.

**Table 9:** Results of Diag-Features model using BraTS2018 and BraTS2020.

Dataset	Accuracy(%)	Recall(%)	Precision(%)
BraTS2018	94.39	90.87	93.54
BraTS2020	94.86	87.56	94.11

### Comparison experiment

In order to highlight the superiority of the proposed algorithm compared to purely data-driven algorithms, we compared the MCE model with some classic baseline models and conducted permutation tests to verify the reliability and stability of the results (Table 10). Meanwhile, we compared the proposed method with recent state-of-the-art methods experimented on BraTS2018 and BraTS2020 datasets (Table 11). From Tables 10 and 11, it can be seen that compared to classical baseline models, the proposed method has outstanding advantages, far surpassing baseline purely data-driven models. Moreover, the permutation test results of the predictive probabilities between the three baseline models and MCE are all less than 0.05, showing significant differences. Additionally, in comparison with recent studies, MCE has also achieved considerable success. The uniqueness of the MCE model lies in its focus on extracting high-quality features to enhance classification performance, rather than just improving classifiers and training strategies. The advantage of this approach is that it can better understand and utilize the information in the input data, achieving outstanding accuracy on the BraTS2018 and BraTS2020 datasets, reaching 96.14% and 97.57%, respectively, surpassing current mainstream classification methods. This is attributed to our improved feature extraction techniques, embedding knowledge features into the feature extraction methods, successfully obtaining high-quality features from small-scale data, effectively enhancing model performance and generalization capability.

**Table 10:** Comparison results with 3D baseline models.

Method	Datasets	Accuracy(%)	Recall(%)	Precision(%)	Permutation test P-value
3D VGG16	BraTS2018	73.68	97.56	73.68	<0.001
	BraTS2020	77.34	93.33	81.16	<0.001
3D GoogleNet	BraTS2018	78.95	97.62	78.85	<0.001
	BraTS2020	74.67	100.00	73.97	<0.001
3D AlexNet	BraTS2018	75.44	97.62	75.93	<0.001
	BraTS2020	74.67	98.15	74.65	<0.001
MCE	BraTS2018	96.14	93.44	97.06	-
	BraTS2020	97.57	92.80	95.96	-

**Table 11:** Comparison results with other recently relevant studies.

Authors	Methods	Datasets	Accuracy (%)
Suárez-García et al. [21]	MLR	BraTS2018	94.12
Rehman et al. [15]	3DCNN	BraTS2018	92.67
Narmatha et al. [16]	GLCM+FBSO	BraTS2018	93.85
Sharif et al. [17]	M3BTCNet	BraTS2018	94.6
Deepa et al. [18]	CNN+DRN+CJHBA	BraTS2018	92.10
Bidkar et al. [19]	SWO-based DBN	BraTS2018	93.3
		BraTS2020	92.1
Jemimma et al. [20]	WCSO-DBN	BraTS2018	92.7
		BraTS2020	94.5
Proposed Approach	MCE	BraTS2018	96.14
		BraTS2020	97.57

### Ablation experiment

In the final phase of our study, we determined the final prediction results by averaging the scores of classifiers trained on deep learning features, Radiomics features, and manual diagnostic features through ensemble voting. To validate the effectiveness of each extracted and manually created features, we conducted four sets of comparative experiments:

1. Classification using deep features extracted by CLNN, Radiomics features and manually created diagnostic features (CLNN+Radiomcs+ Diag);
2. Classification using deep features extracted by MSANN, Radiomics features and manually created diagnostic features (MSANN+Radiomcs +Diag);
3. Classification using deep features extracted by MSANN and CLNN, anually created diagnostic features (CLNN+ MSANN+ Diag);
4. Classification using deep features extracted by MSANN and CLNN, Radiomics features (CLNN+ MSANN+Radiomcs);
5. All (MSANN+CLNN+Radiomcs+Diag).

**Fig 13:**
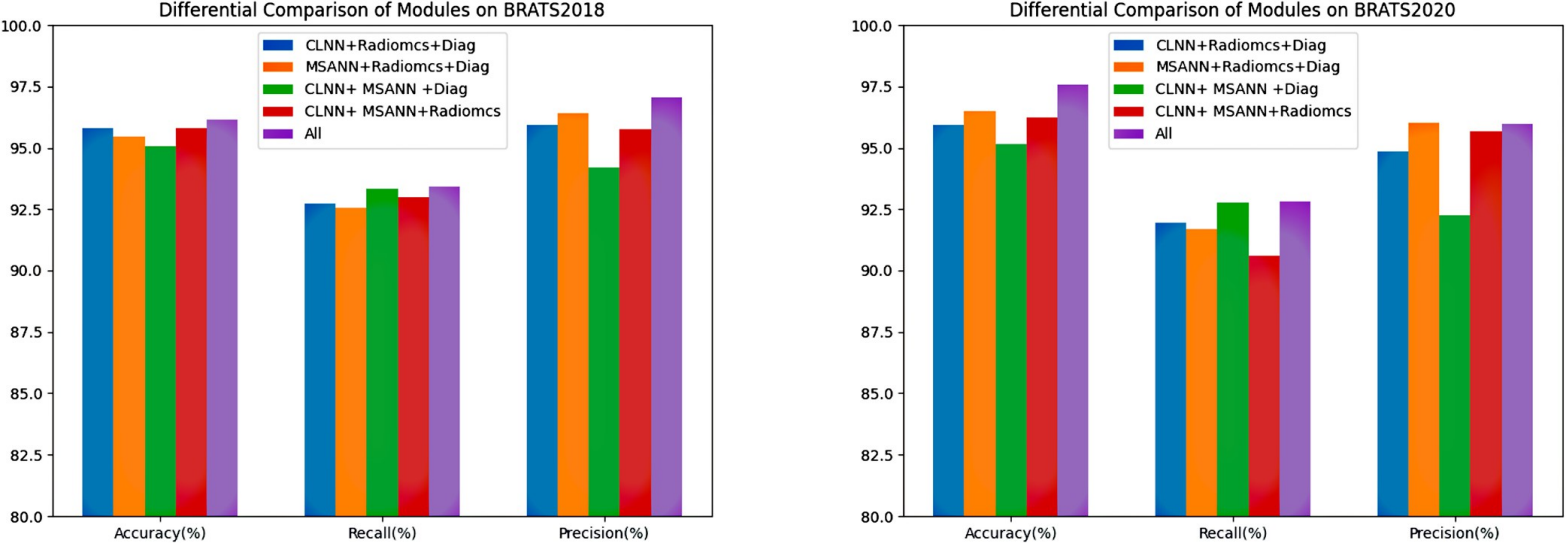
Bar chart depicting the results of ablative experiments for each module in MCE.

We also conducted permutation tests on (1)(5), (2)(5), (3)(5), (4)(5) respectively to accurately demonstrate the effectiveness of the four modules. Table 12 and Fig 13 show the results of the four comparative experiments, and the experiments prove that the various features mined in the method have positive guidance for glioma staging.

**Table 12:** Results of ablative experiments for each module in MCE.

Method	Datasets	Accuracy(%)	Recall(%)	Precision(%)	Permutation test P-value
CLNN+Radiomcs+ Diag	BraTS2018	95.79	92.72	95.95	0.195
	BraTS2020	95.95	91.93	94.85	0.173
MSANN+Radiomcs +Diag	BraTS2018	95.44	92.50	96.39	0.159
	BraTS2020	96.49	91.67	96.03	0.202
CLNN+ MSANN +Diag	BraTS2018	95.08	93.35	94.21	0.041
	BraTS2020	95.14	92.75	92.23	0.024
CLNN+ MSANN+Radiomcs	BraTS2018	95.79	93.00	95.77	0.048
	BraTS2020	96.22	90.62	95.69	0.010
All	BraTS2018	96.14	93.44	97.06	-
	BraTS2020	97.57	92.80	95.96	-

Specifically, from the comparison between Experiment (1) and Experiment (5), it can be observed that the addition of MSANN has a greater impact on improvement in BraTS2020 than in BraTS2018, particularly in terms of accuracy. This could be attributed to the fact that MSANN is centered around deep learning, and issues related to the sample size may influence its effectiveness. Comparing Experiment (2) with Experiment (5), the integration of CLNN significantly enhances the overall performance of the model. Through a curriculum design with progressively challenging tasks and guided interpretation akin to radiological education, the model can better understand rule-based knowledge behind the tasks, leading to more rational feature extraction. Furthermore, from the comparison between Experiment (3) and Experiment (5), the inclusion of Radiomics features results in a substantial increase in accuracy. These features, encompassing texture, morphology, grayscale distribution, among others, prove to be highly beneficial for glioma staging. Lastly, the comparison between Experiment (4) and Experiment (5) shows that manually crafted diagnostic features predominantly driven by NCR/NET and ET region information play a crucial role in post-processing, aiding the model in achieving better classification results in glioma staging.

However, from the results of the permutation tests, it can be seen that the ablation experiments regarding MSANN and CLNN are not very ideal, as the P-values of both are greater than 0.05, indicating no significant differences. This is because their inherent accuracy in classification is much lower than that of Radiomics and Diag, which may result in lower classification contributions. In the ablation experiment results concerning Radiomics and Diag, the P-values of the permutation tests are all less than 0.05, showing significant differences. This demonstrates that the manually extracted features they provide contribute much more to predicting GBM than deep features. This also indirectly indicates that domain expertise is more directly and efficiently helpful for predicting GBM.

## Discussion

The proposed method emphasizes improving the quality of feature extraction, and 4 feature extraction modules were designed for 3D MRI, including MSANN, CLNN, Radiomics Feature Extracter, and Diag-Feature Extracter. MSANN and CLNN were designed at both the model and strategy levels to mimic the radiologist’s image interpretation process in obtaining deep features. In MSANN, the incorporation of CAM and SAM allows the model to emulate the diagnostic attention of a radiologist by focusing on MRI sequences and lesion areas more relevant to the results. This involves assigning more biased weights to regions that are more relevant to the inference results and adaptively overlaying and emphasizing the weights of different image sequences. CLNN, leveraging curriculum learning strategy, mimics the sequential process of a radiologist’s image interpretation and attention to specific regions of different sequences, enabling the model to learn more reasonable features. Radiomics feature extraction from different sequences enables the model to capture information such as texture, morphology, and grayscale distribution specific to different lesion tissues, fully exploiting the roles of different image sequences. Guided by the feature extraction and classification results of imageomics, we found that the ET and NCR/NET regions contain more critical information for glioma staging. Therefore, we devised the Diag-Features primarily focus on these two regions, which has been well-validated through independent two-sample T-tests and Pearson Correlation Coefficient analysis. The success of the MCE model demonstrates the importance of feature extraction in medical image classification and how advanced feature extraction techniques can be utilized to enhance classification performance. This research provides valuable insights for the future development of the field of medical image analysis and offers potential methods and tools for further improving disease diagnosis and treatment.

It should be emphasized that this paper primarily focuses on the enhancement of feature extraction methods through the embedding of knowledge. The goal is to enable the model to incorporate reasonable external rules to guide the feature extraction process. Therefore, minimal selection and adjustment have been applied to the three types of feature classifiers, relying mainly on empirical and experimental insights. Additionally, this method incurs higher computational costs compared to other existing research, primarily due to its specialized design for 3D data. Nevertheless, in the context of glioma staging diagnosis, we believe that reliability should take precedence over considerations such as time and computational cost.

## Conclusions

Medical imaging plays a crucial role in medical diagnosis, with continuous advancements propelled by innovations in image processing techniques. The emergence and evolution of deep learning technology have undoubtedly been one of the key drivers of advancements in medical image analysis in recent years. However, the scarcity of brain glioma MRI data makes it challenging for purely data-driven deep learning algorithms to extract high-quality features and capture their complex patterns, significantly affecting generalization capability. In order to extract higher-quality features from a limited number of samples, this paper introduces the MCE model, which incorporates external knowledge rules into the feature acquisition process. Furthermore, to address the issue of selecting the optimal slice when analyzing 2D slices, our model is meticulously designed based on 3D MRI data for brain imaging. We have designed two feature extraction models, MSANN and CLNN, to emulate the attention patterns of medical professionals when reviewing medical images. Additionally, we have designed an Radiomics features extraction model to capture specific information such as image texture, morphology, and grayscale distribution. Moreover, we have also designed manually created diagnostic features based on the results of Radiomics experiments, key points in radiological diagnosis, and imaging characteristics of the image sequences. Through these improvements in feature extraction, we have successfully achieved the high-quality features with increased information density. By introducing knowledge characteristics and external rules, model effectively incorporates the knowledge and expertise of medical professionals. Experimental results demonstrate that our proposed method outperforms purely data-driven models, achieving significant improvements in the classification and staging of brain gliomas. On the BraTS2018 and BraTS2020 datasets, our method achieves impressive Accuracy, Recall, and Precision rates of 96.14%, 93.44%, and 97.06%, as well as 97.57%, 92.80%, and 95.96%, respectively.
